# The Italian version of the Reflective Functioning Questionnaire: Validity data for adults and its association with severity of borderline personality disorder

**DOI:** 10.1371/journal.pone.0206433

**Published:** 2018-11-01

**Authors:** Niccolò Morandotti, Natascia Brondino, Alessia Merelli, Annalisa Boldrini, Giulia Zelda De Vidovich, Sara Ricciardo, Vera Abbiati, Paolo Ambrosi, Edgardo Caverzasi, Peter Fonagy, Patrick Luyten

**Affiliations:** 1 Interdepartmental Center for Research on Personality Disorders, Department of Applied and Behavioral Sciences of Psychiatry, University of Pavia, Pavia, Italy; 2 Research Department of Clinical, Educational and Health Psychology, UCL, London, United Kingdom; 3 Faculty of Psychology and Educational Sciences, KU Leuven, Leuven, Belgium; University of La Rioja, SPAIN

## Abstract

**Introduction:**

Impairments in the ability to understand others and the self in terms of internal mental states (reflective functioning [RF] or mentalizing) are thought to play a key role in the development of borderline personality disorder (BPD). The first aim of this study was to validate the Italian version of the Reflective Functioning Questionnaire (RFQ), a brief self-report measure of RF, by examining its factor structure with Principal Component Analyses (PCA), and correlations with constructs that should be theoretically related to RF. In addition, we investigated whether the RFQ could empirically distinguish between healthy controls and carefully diagnosed BPD patients using Research Operating Curve methods, and was related to severity of borderline pathology as measured with the Shedler–Westen Assessment Procedure (SWAP), an observer-rated measure of BPD pathology.

**Methods:**

An Italian translation of the RFQ was administered to a sample of 154 healthy controls and a clinical sample of 59 BPD patients diagnosed with the Structured Clinical Interview for DSM-IV Axis II disorders. Clinical severity of BPD was assessed with the SWAP. Normal controls completed self-report inventories of constructs related to RF (mindfulness, empathy, Theory of Mind, alexithymia, and autistic traits).

**Results:**

PCA confirmed the a priori factor structure in the Italian translation of the RFQ, showing two subscales that measure certainty and uncertainty about mental states, with satisfactory reliability and construct validity. These dimensions also distinguished BPD patients from healthy controls (*p* < 0.05). ROC analyses showed that the uncertainty subscale discriminated BPD patients from healthy individuals (area under the curve = 78%, cut of 4.5 points, sensitivity = 73%, specificity = 68%). Within the patient group, regression analyses showed uncertainty about mental states to have a significant unique contribution in predicting BPD severity (*p* < 0.05), explaining 12% of the variance.

**Conclusions:**

Results largely supported the reliability and validity of the Italian version of the RFQ. These findings also provide further evidence for the role of impairments in mentalizing and reinforce the rationale for offering mentalization-based interventions to individuals with this disorder.

## Introduction

Borderline personality disorder (BPD) is a psychiatric disorder characterized by instability in self-image and interpersonal relationships, impairments in emotion regulation, and impulsivity, which may lead to disruptive and self-harming behaviors [1]. The prevalence of BPD in general population has been reported to be 5.9% in the United States of America [2]. The disorder has been associated with high levels of subjective distress, social disability, and intensive utilization of mental health services [3]. Despite the impact of borderline pathology on global functioning and the high burden of the disease, BPD is often misdiagnosed due to its high psychiatric comorbidity and to the inability of diagnostic measures to assess its underlying factors [2, 4, 5].

The mentalizing approach to BPD [6–9] highlights how impaired reflective functioning (RF) mediates borderline symptoms. RF, or mentalizing, refers to the ability to understand others and the self in terms of internal mental states, such as beliefs, desires, feelings and attitudes [10]. High levels of RF allows affect regulation, the development and maintenance of a robust sense of self, and constructive social interactions [11]. The ability to mentalize first develops in the context of early attachment relationships; BPD patients have been found to often have disorganized and preoccupied attachment styles [12, 13], reflecting poor quality of attachment relationships in many of these individuals’ childhood [14, 15]. While many studies have provided evidence for the role of impairments in mentalizing in BPD [16–20], to our knowledge, no study in Italy has explored the relationship between poor mentalizing and BPD.

RF has historically been measured with the Reflective Functioning Scale (RFS) [21], an instrument that evaluates RF on the basis of rating narratives elicited by the Adult Attachment Interview [22] about interviewees’ childhood attachment experiences and the way these have affected their present-day views of themselves and their close relationships. Although the RFS remains the gold standard method of assessing RF, it lacks feasibility due to the considerable amount of time needed to administer and score it, and it also requires highly trained administrators/scorers. Furthermore, both clinical and research contexts require a tool to assess RF that could be easily administered and repeated across time to investigate treatment outcomes in large samples. Previous studies have employed validated self-report inventories measuring constructs that overlap with RF, such as mindfulness, metacognition, and empathy. However, such measures provide only an indirect assessment of RF [23, 24], and the constructs above mentioned only partially overlaps with mentalization. Metacognition has been found to be impaired in BPD [25] and can be measured with different assessment tools, such the Self-Report measure of Metacognition [26] and the Metacognition Assessment Interview [27]. However, although it refers to skills that allow people to recognize mental states, it considers mind reading as a general ability generated by the interaction of different components [27].

To overcome these limitations, Fonagy and colleagues recently validated the first self-report measure of RF, the Reflective Functioning Questionnaire (RFQ) [28]. The RFQ has two subscales, which assess certainty (RFQ_C) and uncertainty (RFQ_U) about the mental states of self and others. High scores on these subscales indicate two distinct impairments in RF, respectively, hypomentalizing and hypermentalizing: hypomentalizing reflects concrete thinking and poor understanding of the mental states of self and others, while hypermentalizing refers to an “over-mentalizing” attitude, that is, developing too certain and detailed models of the mind and mental states not supported by the evidence.

### Study hypotheses

The aims of the present study are the validation of the Italian version of the RFQ and the investigation of its ability to distinguish between healthy individuals and BPD patients. Indeed, translation and transcultural validation into Italian are urgently needed to facilitate research in the mentalization field among Italian-speaking populations. In line with previous work [24, 28] we expected to find evidence for a two-factor structure, reflecting levels of subjective uncertainty or certainty about the link between behaviors and mental states. We then investigated the internal consistency and test–retest reliability of the RFQ subscales, and their relationships with demographic variables. The convergent and divergent validity of the RFQ was investigated by studying the correlations between the RFQ and measures of concepts theoretically related to RF [23, 29] in healthy participants.

Positive associations between the degree of certainty about mental states (RFQ_C) and mindfulness, empathy, and Theory of Mind capacity were expected; however, correlations with Theory of Mind were expected to be relatively lower, in that this construct refers to mentalizing based on external features, while the RFQ is primarily designed to test individuals’ ability to focus on internal mental states rather than external physical cues. Negative correlations between RFQ_C and alexithymia and autistic traits were expected, in that these dimensions indicate poor awareness of emotions or feelings and “mind-blindness”. Correlations between the degree of uncertainty about mental states (RFQ_U) and the constructs listed above were hypothesized to show the opposite pattern. The discriminant validity of the RFQ was established by testing its ability to differentiate between healthy and BPD participants.

Further, having established the validity of the Italian version of the RFQ, on the basis of the literature about mentalizing, we hypothesized that impairments in RF measured with the RFQ would be related to the severity of borderline pathology in BPD patients.

## Methods

### Participants and clinical assessment

RFQ data were collected in samples of BPD patients and healthy individuals. Inclusion criteria for all participants were age between 18 and 50 years and being a native Italian speaker. Participants were considered eligible for the analyses provided there were not too many missing values (more than 5% of missing data); outliers were removed from the analyses if the error checking was not successful.

For the clinical BPD sample, 62 patients fulfilling criteria for BPD based on the fourth edition of the *Diagnostic and Statistical Manual of Mental Disorders* (DSM-IV) [1] were recruited between November 2011 and November 2015 from the Center for Research on Personality Disorders of the University of Pavia, Italy, an outpatient unit dedicated to the study and treatment of personality disorders in patients referred by community mental health services. In order to ensure representativeness of clinical practice, exclusion criteria were restricted to a stable current or past history of psychotic disorders, personality disorder due to medical conditions, antisocial personality disorder, and severe current drug addiction [28]. Patients were assessed by a specialist psychiatrist using the Italian versions of the Structured Clinical Interview for DSM-IV Axis I disorders (SCID-I) [30, 31] and the Structured Clinical Interview for DSM-IV Axis II disorders (SCID-II) [32, 33]. BPD severity was measured with the Shedler–Westen Assessment Procedure (SWAP-200) [34, 35]. Measurements of anxiety and depression were obtained by using the Italian versions of the Hamilton Anxiety Scale [36, 37] and the Hamilton Depression Rating Scale [38, 39], respectively. Inter-rater reliability was excellent for all assessment scales (Cohen’s k > 0.75) [40].

Of the 62 BPD patients, 2 were excluded because of missing values (response rate: 96.7%); 1 was excluded as outlier. Between the remaining 59 patients, 42 had one or more current comorbid Axis I or Axis II diagnosis (depressive disorder = 22, panic disorder = 5, alcohol or drug abuse = 6, dependent personality disorder = 8, avoidant personality disorder = 4). Twenty-nine patients were medicated; 18 were on antidepressants (13 on selective serotonin reuptake inhibitors, 5 on serotonin noradrenalin reuptake inhibitors,); 9 on mood stabilizers (7 on valproic acid, 2 on oxcarbazepine); 12 were on atypical antipsychotics (9 in combination with an antidepressant, 1 in combination with a mood stabilizer, 2 on monotherapy).

As a comparison group, 158 healthy participants were recruited via advertisements at the University of Pavia; these individuals comprised students and service staff. 4 of them were excluded because of missing values (response rate: 97.5%). See Table 1 for details of the demographic characteristics of the sample. All participants were Caucasian.

**Table 1:** Demographic characteristics of borderline personality disorder (BPD) patients and healthy participants. NS = not significant (*p* > 0.05).

	Healthy participants	BPD patients	Significance
Participants (*n*)	154	59	–
Sex (male %)	42	29	NS
Age (mean [SD])	26.6 (6.1)	29.1 (8.5)	NS
Years of education(mean [SD])	16.4 (2.2)	15.2 (3.5)	t = 2.2 *p* < 0.05
Marital status (*n*)			
Single	147	50	χ2(1) = 7.04, *p* < 0.01
Married	7	9	

All participants gave written informed consent in accordance with the requirements of the ethics committee of the Department of Applied and Behavioral Sciences of Psychiatry, University of Pavia, Pavia, Italy, which approved this study.

### Measures

The RFQ and the self-report measures of constructs related to RF were individually administered to the healthy participants. Participants were supervised by a trained psychiatrist while completing the measures to ensure appropriate understanding of items.

#### RFQ

As first step of the validation process, the RFQ was translated into Italian by a native Italian speaker; to maintain sentences’ meaning between English and Italian, the Italian version of the RFQ underwent a back-translation [41]. To this end, the Italian RFQ was translated back to English by a native English speaker naïve to the RFQ; this version was compared to the original version of RFQ. Discrepancies were corrected. The International Test Commission Guidelines for test translation and adaptation [42] were checked to ensure proper questionnaire adaptation across different cultures.

The RFQ comprises two subscales, each containing 6 items, measuring the degrees of uncertainty (RFQ_U) and certainty (RFQ_C) about mental states. The RFQ_U subscale consists of 6 items focusing on the level of participant agreement, with statements such as “Sometimes I do things without really knowing why”. Items are scored by the participant on a 7-point Likert scale (ranging from “completely disagree” to “completely agree”). To capture extreme levels of uncertainty, items are rescored to 0, 0, 0, 0, 1, 2, 3, so that very high agreement on this scale reflects hypomentalizing (i.e. a lack of knowledge about mental states), while some disagreement reflects adaptive acknowledgement of the opaqueness of one’s own mental states, typical of genuine mentalizing. The RFQ_C subscale consists of 6 items, such as “I don’t always know why I do what I do”, and is scored on the same 7-point Likert scale as the RFQ_U, then rescored to capture extreme levels of certainty; to this end, these items are recoded to 3, 2, 1, 0, 0, 0, 0. As a result, low agreement reflects hypermentalizing, while some agreement reflects adaptive levels of certainty about mental states.

#### Reading the Mind in the Eyes Test

The Reading the Mind in the Eyes Test (RMET) [43, 44] was used to assess Theory of Mind. In this instrument, individuals choose one out of four adjectives describing the state of mind of 36 individuals, of whom they see pictures of only the eye region. The score on the RMET derives from the sum of correct answers.

#### Empathy Quotient

The Empathy Quotient (EQ) [45, 46] was administered to measure levels of empathy. The EQ includes 40 questions rated on a 4-point Likert scale ranging from “strongly agree” to “strongly disagree”. The total score can range from 0 to 80. Internal reliability in the healthy participants sample (Cronbach’s α = 0.81) was excellent.

#### Kentucky Inventory of Mindfulness Skills

The Kentucky Inventory of Mindfulness Skills (KIMS) [47] was administered to evaluate participants’ ability to focus their attention on what is taking place in the present with a non-judgmental attitude. The KIMS is a 39-item inventory, developed in order to measure four aspects of mindfulness (observing, describing, acting with awareness and accepting without judgment) on a 5-point Likert scale, ranging from 1 (“very rarely true”) to 5 (“very often true”). In this study, only the Acting with Awareness (KIMSac) subscale was used. Internal reliability in the healthy participants sample (α = 0.78) was excellent.

#### Toronto Alexithymia Scale

The Toronto Alexithymia Scale (TAS-20) [48, 49] comprises 20 statements rated on a 5-point Likert scale. It includes three subscales which measure three facets of alexithymia, such difficulties in identifying feelings, difficulties in describing feelings and lack of focus on internal emotional experiences. For this study, the total TAS-20 score was used. Internal reliability in the healthy participants sample (α = 0.82) was excellent.

#### Autism Spectrum Quotient

The Autism Spectrum Quotient (AQ) [50, 51] was employed to measure autistic traits. The AQ has 50 forced-choice questions (rated on a 4-point Likert scale ranging from “strongly agree” to “strongly disagree”) that allow to quantify autistic traits across domains of social skills, imagination, communication, attention to detail and attention switching. Participants score one point for each question they answer as autistic people. Total scores (ranging from 0 to 50) were used. Internal reliability in the healthy participants sample (α = 0.76) was good.

#### Shedler–Westen Assessment Procedure

The SWAP 200 [34] is an assessment instrument designed to harness clinical judgment and inference, in order to systematize clinical observation. It consists of a set of 200 statements, each of which can characterize a given patient not at all, somewhat, or very well. Statements are clinically comprehensive and can be used by any skilled clinical observer, irrespective of his theoretical orientation. The patient is described by the clinician by ordering the statements into eight categories; those that are most descriptive are assigned a value of 7, while those that are not at all descriptive are assigned a value of 0. Consequently, a score from 0 to 7 can be assigned to each of 200 personality descriptive variables. The SWAP 200 vocabulary permits clinicians to obtain accurate psychological descriptions of patients in a quantifiable form. The SWAP scoring algorithms provide a dimensional score for each personality disorder included in the DSM IV. Dimensional scores measure the similarity between a given patient and prototypical SWAP descriptions symbolizing each personality disorder in its characteristic form. Inter-rater reliability between independent interviewers has been above 0.80 for all SWAP diagnostic scales.

In the present study, to optimize the power of clinical observations, SWAP scores were performed by the psychiatrist that clinically assessed each BPD patient.

### Data analysis

Principal component analysis (PCA) was performed to study the factor loading of both subscales of the RFQ.

The reliability of the subscales was estimated by calculating Cronbach’s alpha coefficients and mean inter-item correlation. Test–retest correlation after an interval of 2 weeks was calculated in a subsample (*n* = 30) of healthy participants, in order to demonstrate temporal stability.

Pearson correlations between the RFQ subscales and both demographics and clinical features were explored.

To investigate the construct validity in the sample of healthy individuals, Pearson correlations between the RFQ subscales and measures of related constructs were explored. Bonferroni correction was applied.

The discriminant validity of the RFQ was investigated by performing analyses of covariance (ANCOVA) in the whole sample, with the RFQ subscale scores entered as dependent variables, with diagnosis as a factor and age as a covariate. Receiver Operating Curve (ROC) analysis was performed to investigate the ability of the RFQ_C and RFQ_U subscales to identify clinical cases, and to determine a cut-off score.

Hierarchical multiple regressions were conducted in the clinical sample with the SWAP-200 borderline score as dependent variable, and age, depression and RFQ_U scores as independent variables. An inverse relationship between age and BPD severity [52] and a positive correlation between age and RF [28] has previously been reported. Moreover, comorbidity between depressive disorders and BPD is very common [53]. Anxiety was not entered into the analyses due to its collinearity with depression.

SPSS 17.0 (SPSS Inc., Chicago, IL, USA) was used for analysis of demographics and the psychological and psychopathological measures.

## Results

### Principal component analysis

The RFQ_C and RFQ_U subscale items were subjected to PCA using data from the sample of healthy participants. First, the suitability of the data for factor analysis was assessed. The Kaiser-Meyer-Olkin value was 0.8 for RFQ_U and 0.76 for RFQ_C; Bartlett’s test of sphericity reached statistical significance (*p* < 0.05) for both RFQ_U and RFQ_C, supporting the factorability of the data.

Inspection of scree plots revealed for both RFQ_U and RFQ_C the presence of one component with an eigenvalue > 1. The one-component solutions explained a total of 47.0% of variance in RFQ_U scores and 46.3% of variance in RFQ_C scores. Between items correlations and factor loadings are reported below (Tables 2 and 3).

**Table 2:** Correlations between items of certainty subscale (RFQ_C) and uncertainty subscale (RFQ_U); cs stands for certainty scale items, us for uncertainty scale items.

		**cs1**	**cs16**	**cs20**	**cs36**	**cs40**	**cs 44**
**RFQ_C**	**cs1**	1.000	0.457	0.303	0.225	0.350	0.333
	**cs16**	0.457	1.000	0.412	0.282	0.270	0.598
	**cs20**	0.303	0.412	1.000	0.623	0.412	0.461
	**cs36**	0.225	0.282	0.623	1.000	0.334	0.370
	**cs40**	0.350	0.270	0.412	0.334	1.000	0.338
	**cs44**	0.333	0.598	0.461	0.370	0.338	1.000
		**us16**	**us28**	**us36**	**us40**	**us44**	**us8**
**RFQ_U**	**us16**	1.000	0.422	0.383	0.339	0.582	0.417
	**us28**	0.422	1.000	0.532	0.451	0.421	0.352
	**us36**	0.383	0.532	1.000	0.287	0.409	0.292
	**us40**	0.339	0.451	0.287	1.000	0.430	0.427
	**us44**	0.582	0.421	0.409	0.430	1.000	0.419
	**us8**	0.417	0.352	0.292	0.427	0.419	1.000

**Table 3:** Factor loadings for RFQ subscales items.

	RFQ_C	RFQ_U
RFQ item	Factor 1	Factor 1
**cs1**	0.781	
**cs16**	0.754	
**cs20**	0.729	
**cs36**	0.679	
**cs40**	0.626	
**cs44**	0.615	
**us16**		0.772
**us28**		0.745
**us36**		0.742
**us40**		0.678
**us44**		0.672
**us8**		0.669

### Reliability

The Italian RFQ subscales had good internal consistency, with Cronbach’s alpha coefficient of 0.77 for RFQ_U and 0.75 for RFQ_C. Mean inter-item correlations were 0.36 and 0.35, respectively. The test–retest reliability was good, with *r* = 0.85 for RFQ_U and *r* = 0.81 for RFQ_C (both *p* < 0.001).

### Correlations between RFQ scores and demographic, psychological and clinical variables

Consistent with expectations, RFQ_C was positively correlated with measures of mentalizing-related abilities—the KIMSac, the EQ, and the RMET (see Table 4). The correlation with the RMET was lower, in accordance with expectations, and was not significant after Bonferroni correction. The RFQ_C was inversely correlated with the TAS and AQ, as expected. Correlations between RFQ_U and mentalizing-related constructs showed the opposite pattern to those observed for RFQ_C. No significant correlation was found between the RFQ subscales and demographic variables.

**Table 4:** Significant correlations between certainty (RFQ_C) and uncertainty (RFQ_U) about mental states and measures of mentalizing-related constructs among healthy controls (*n* = 154).

	RFQ_C	RFQ_U	TAS	AQ	EQ	KIMSac
**RFQ_C**		*r* = -0.671*	*r* = -0.530*	*r* = -0.223*	*r* = 0.395*	*r* = -208*
**RFQ_U**	*r* = -0.671*		*r* = 0.431*	*r* = 0.251*	*r* = -0.224*	*r* = 0.276*

Note: TAS = Toronto Alexithymia Scale; AQ = Autism Quotient; EQ = Empathy Quotient; KIMSac = Kentucky Inventory of Mindfulness Skills Acting with Awareness subscale.

* = *p* < 0.01

### Group differences

The ANCOVAs showed a significant effect of diagnosis (BPD vs. controls) on RFQ_C and RFQ_U scores (RFQ_C: *F*_1,210_ = 7.1, *p* < 0.01, partial eta squared = 0.12; RFQ_U: *F*_1,210_ = 55.6, *p* < 0.001, partial eta squared = 0.32).

**Fig 1:**
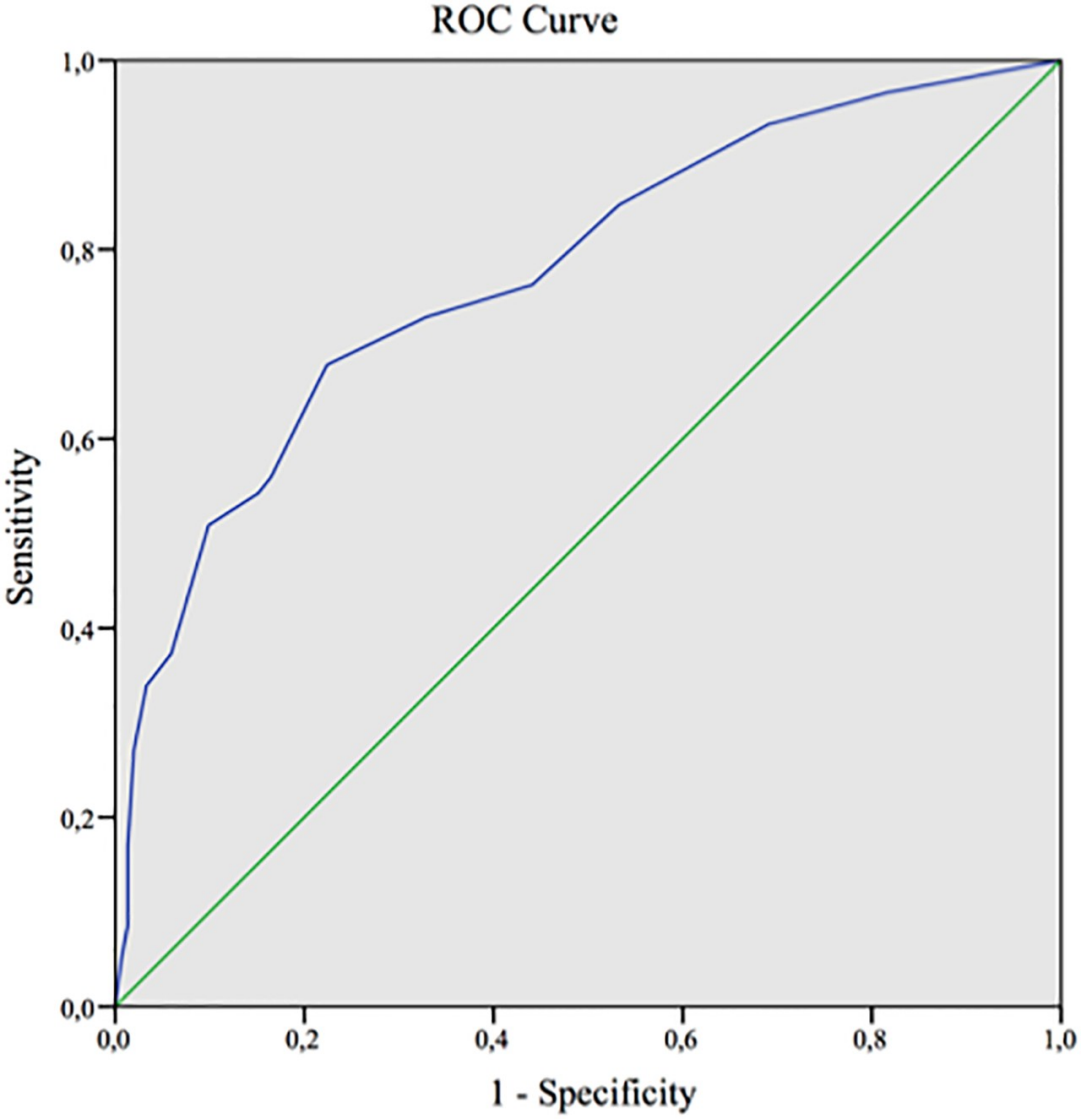
Receiver operating characteristic curve using RFQ_U to discriminate between healthy participants and borderline personality disorder patients.

ROC analyses showed that the RFQ_U subscale discriminated BPD patients from healthy controls (area under the curve = 0.78%, cut-off 4.5 points, sensitivity = 73%, specificity = 68%) (Fig 1).

### Effect of impaired mentalizing on severity of BPD pathology

RFQ_U scores were correlated with severity of BPD (see Table 5). Regression analyses were performed to explore the unique contribution of uncertainty about mental states to severity of BPD pathology. The first step of the hierarchical multiple regression showed no significant effect of age and depression on severity of BPD measured with the SWAP-200. These variables explained 11.5% of the variance in BPD severity, but the model was not significant. However, after entering RFQ_U as an independent variable in the second step of the analysis, the total variance explained by the model was 20.3% (*F*_3,39_ = 3.3, *p* < 0.05). This indicated that uncertainty about mental states explained an additional 8.8% of the variance in BPD severity as measured with SWAP-200, after controlling for age and depressive symptoms (*R*^2^ change = 0.088, *F* change (1,39) = 4.3, *p* < 0.05). In the final model, only RFQ_U was statistically significant (β = 0.34, *p* < 0.05).

**Table 5:** Correlations between uncertainty (RFQ_U) and certainty (RFQ_C) about mental states and measures of depression, anxiety, age and severity of borderline personality disorder (BPD) in a sample of 59 BPD patients.

	RFQ_C	RFQ_U	HAS	HDRS	Age	SWAP-200
RFQ_C		*r* = -0.76*	*r* = 0.39*	*r* = 0.35*	*r* = 0.20	*r* = -0.15
RFQ_U	*r* = -0.76*		*r* = -0.06	*r* = -0.24	*r* = -0.20	*r* = 0.23**

Note: HAS = Hamilton Anxiety Scale; HDRS = Hamilton Depression Rating Scale; SWAP-200 = Shedler-Westen Assessment Procedure for BPD.

* = *p* < 0.01

** = *p* < 0.05

## Discussion

This study first aimed to validate the factor structure of the Italian version of the RFQ by factor analysis and by examining its correlations with related constructs and clinical variables in a sample of healthy individuals. Furthermore, this study also investigated the ability of the RFQ to distinguish between healthy controls and BPD patients. A final aim was to investigate the relationship between impairments in RF as measured with the RFQ and the severity of BPD psychopathology in a sample of BPD patients.

PCA resulted in the extraction of single factors for both the RFQ_U and RFQ_C subscales, reflecting the conceptualization of the original version of the RFQ [28]. Both subscales had good internal consistency and reliability.

The RFQ subscales were also in theoretically expected ways related to constructs that are closely related to RF, such as empathy and aspects of mindfulness. These results are consistent with mentalizing theory, in that empathy and mindfulness, like RF, imply the capacity for internally-based mentalizing. The construct of empathy, although broad and complex, involves the ability to detect other people’s mental states and predict their future behavior [54], and therefore partly overlaps with the construct of RF. Correlations between the RFQ subscales and Theory of Mind were also in the expected direction, although they were lower as expected, and even not statistically significant. This provides further support for the assumption that the RFQ primarily taps into internally-based mentalizing, while Theory of Mind involves mentalizing about external features of others (i.e., facial expressions), a feature of mentalizing that is relatively distinct from internally-based mentalizing [44, 29].

Negative correlations were found between the RFQ_C subscale and the constructs of alexithymia and symptoms of autistic spectrum disorder; conversely, positive relations were found between the RFQ_U subscale and these same constructs. Indeed, alexithymia, that is, the inability to experience, identify and express emotions, reveals uncertainty about the emotional states of others and the self, and, as such, poor RF. Likewise, the presence of autistic traits implies poor imaginative, communication and social skills—skills that are required attributes of a good reflective capacity.

Both the RFQ_C and RFQ_U were able to differentiate healthy participants from carefully diagnosed BPD patients. The ROC analysis indicated that the RFQ_U subscale was able to discriminate BPD patients from healthy controls, allowing identification of a cut-off, with relatively good sensitivity and specificity for such a brief measure, although of course improvement is possible. The same was not found for the RFQ_C subscale in the ROC analysis, although BPD patients differed significantly from healthy controls on this subscale.

With regard to the relationship between RF and severity of borderline psychopathology, uncertainty about mental states was found to have a unique contribution to the severity of BPD as independently assessed by trained observers using the SWAP. These results are consistent with mentalizing approaches to BPD [6, 9, 11], as they assume that the poorer mentalizing is, the more problems with regard to affect dysregulation, interpersonal instability and impulsive behavior can be expected. The hypersensitivity of BPD patients to their own and others’ emotions [55] results in difficulty in developing plausible scenarios about the inner mental states of the self and others, and difficulty in considering alternative explanations about these features, in turn, are the basis of the difficulties in affect regulation, impulsive behaviors and relationship problems that are characteristic of people with BPD.

A strength of this study is that all participants BPD were recruited directly from a clinical population drawn from a clinical setting specialized in the treatment of BPD. However, some methodological limitations must be mentioned. First, our analyses and conclusions are largely based on self-report questionnaires, with the exception of the SWAP. A further weakness is the lack of an appropriate criterion for measuring mentalizing; therefore, our conclusion that the RFQ actually measures RF can be only provisional. A further limitation is the fact that participants’ emotional state at the time of assessment may have had an impact on the results. Indeed, Fonagy et al. [6] point out that mentalizing may be in part context-specific. Another important limitation is the comorbidity of BPD with other psychiatric disorders, particularly major depression. However, the frequency of psychiatric comorbidity in BPD patients is notoriously high in clinical practice [53]; excluding individuals from the present study because of comorbidity could have led to the selection of a non-representative sample, thereby limiting the generalizability of the results. Finally, the socio-economic status of participants was not measured, so it may be the case that healthy individuals and BPD patients differed for this variable.

In conclusion, this study provides preliminary evidence for the reliability and validity of the Italian version of RFQ as a self-report tool for measuring RF. The RFQ_U subscale in particular had good discriminative capacity, but further research is needed to determine whether this instrument can be used as a screening tool for the diagnosis of BPD. However, the RFQ provides a measure of individuals’ reflective abilities and as such it can be used to enhance the clinical assessment of BPD patients, as well as to measure the response to clinical intervention, for example, mentalization-based treatment [24, 28]. Use of the RFQ could also facilitate an increase in the body of research and knowledge about mentalizing. Further studies are needed to investigate the relation between self-report assessments of reflective function and metacognition; indeed there is an increasing amount of research about the measurement of metacognition [25, 26].

## Supporting information

S1 Dataset (XLSX)
